# Advanced nurse practitioners- the missing link on old age pyschiatry inpatient wards?

**DOI:** 10.1192/bjo.2021.869

**Published:** 2021-06-18

**Authors:** Grace Lydia Goss, Priya Gowda, Danika Rafferty

**Affiliations:** 1 Aneurin Bevan University Health Board; 2 Cwm Taf Morgannwg University Health Board

## Abstract

**Aims:**

1. This project aimed to review the medical cover available to an Old Age Psychiatry inpatient ward.

2. To discuss with ward staff their view for potential improvements and areas of clinical development.

3. To review the potential of a Full Time Nurse Practitioner role on the ward.

One junior doctor (CT1 or equivalent) was allocated to cover the ward whilst balancing their other training needs and clinical commitments. The inpatient ward was based in a community hospital with no onsite medical team. The patients mostly had complex medical needs and multiple comorbidities.

**Method:**

The Junior Doctor's timetable and the time allocated to the ward was reviewed. Questionnaires were conducted with nursing staff to assess their views on the support of physical health cover. The patient notes were analysed for the time taken to review patients after falls over a one month period.

**Result:**

There were 14.5hours allocated to ward cover. An additional 4 hours was provided by another visiting junior doctor totalling 18.5hours per week- 11% of the time. This figure does not account for annual leave, on call commitments or study days whereby there was no additional cover.

A short survey completed by ward staff showed- (1 = Very Poor/Difficult 5 = Excellent/Easy)

- They rated medical cover of physical health needs on ward 7 as 1.3.
- They found contacting a Doctor to discuss a physical problem as 1.7- with particular concern for OOH.
- It was rated to be extremely difficult for a same day review of physical health problems- 1.7
- It was rated extremely difficult to get a physical review following a fall on ward 7- 1.4
- Continuity of care for the patients on ward 7 was rated as 1.6.

The patient case files reviewed over a one month period showed x8 falls. These took on average 14 hours before having a review.

**Conclusion:**

Medical cover for the old age psychiatry inpatient ward was inconsistent and a challenge for a single trainee to manage alongside their other clinical commitments and training needs. A case was proposed to management with an SBAR for a Full Time Advanced Nurse Practitioner which has been approved. This role should provide patients with appropriate cover of their physical health needs. It will allow the junior doctor to work alongside them on the ward supporting each other to provide optimal care for the inpatients.

